# Management of a huge right atrial thrombus in a patient with multiple comorbidities

**DOI:** 10.1186/s43044-020-00112-x

**Published:** 2020-11-11

**Authors:** Peter Philip Shaker Selwanos, Ahmed Osman Ahmed, Karim Mohamed El Bakry, Ahmed Nazmy Elsharkawy, Omar Alaaeldin Mohamed, Hatem Hosny, Amir Anwar Shaker Samaan

**Affiliations:** 1 Department of Cardiology, Aswan Heart Centre, Magdi Yacoub Foundation, 72 Kasr Elhagar street, Elsail Elegedeed, Aswan, PO 81511 Egypt; 2 Department of Cardiology, Cairo University Hospital, Cairo, Egypt; 3 Department of Intensive Therapy Unit, Postoperative Cardiac Surgery, Aswan Heart Centre, Magdi Yacoub Foundation, Aswan, Egypt; 4 Department of Cardiac Surgery, Aswan Heart Centre, Magdi Yacoub Foundation, Aswan, Egypt

**Keywords:** 2D echocardiography, Right atrial thrombus, Paradoxical embolism

## Abstract

**Background:**

Floating right heart thrombi (RHT) represent an underdiagnosed, potentially hazardous, and to some extent rare phenomenon in patients presenting with acute pulmonary embolism (APE). Emergent treatment is usually required for such a condition.

**Case presentation:**

A 19-year-old young lady presented with progressive shortness of breath, marked renal impairment, thrombocytopenia, and a highly oscillating huge right atrial mass. After she was admitted to the intensive care unit, she arrested in asystole and was resuscitated, and her electrocardiogram (ECG) showed evidence of acute anterior myocardial infarction. Urgent cardiac surgery to remove the right atrial mass was proposed by the heart team as the best option of management. Surgery was emergently performed with extra-corporeal membrane oxygenator (ECMO) as a support. Following surgery, mechanical support and vasopressors were successfully weaned and the patient achieved a good recovery.

**Conclusions:**

A pulmonary embolism response team (PERT) approach should always be considered where a multidisciplinary team involving a cardiologist, radiologist, cardio-thoracic surgeon, radiologist, and intensivist shall determine the management strategy for a challenging presentation of a massive pulmonary embolism or floating right heart thrombi causing the hemodynamically unstable clinical condition.

**Supplementary Information:**

The online version contains supplementary material available at 10.1186/s43044-020-00112-x.

## Background

Floating right heart thrombi (RHT) probably represent an extreme medical emergency in the context of acute pulmonary embolism (APE) [1]. The prevalence of RHT in the setting of PE is 4–18% [2].

Paradoxical embolism is one of the dreadful complications of RHT. Patent foramen ovale (PFO) has been reported to be an important risk factor for paradoxical systemic emboli. In addition, Several reports have associated acute myocardial infarction with paradoxical embolism.

## Case presentation

A 19-year-old young lady presented to our cardiology clinic complaining of shortness of breath of 3 months duration. Her symptoms were progressive over time, and on presentation, she was short of breath on minimal effort. She also reported two attacks of hemoptysis, and each was about a cup of fresh blood. She denied any history of chest pain, palpitations, syncope, or lower limb swelling.

Six years earlier, she was admitted with generalized anasarca, rapidly deteriorating renal functions, and nephrotic range of proteinuria. Renal biopsy showed focal segmental glomerulosclerosis. She had two sessions of hemodialysis and was kept on immune-suppressive medical therapy, and her renal functions were then stabilized. Furthermore, 2 years before presenting to us, she had a purpuric eruption and was found to have severe thrombocytopenia. Her platelets reached 10,000, and after exclusion of all other possible causes, she was diagnosed with immune thrombocytopenic purpura (ITP) and was kept on steroids. She also reported a history of previous right femoral deep venous thrombosis 1 year ago for which she was anticoagulated using warfarin for 6 months. The patient denied any history suggestive of an autoimmune disorder (e.g., arthralgia, skin rash, mouth ulcers, hair loss), and there was no family history of any similar conditions.

**Fig. 1:**
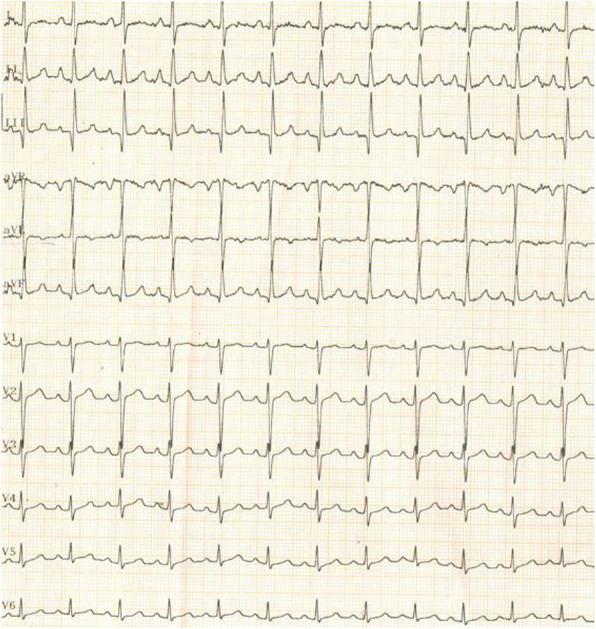
Twelve lead ECG showing sinus tachycardia

**Fig. 2:**
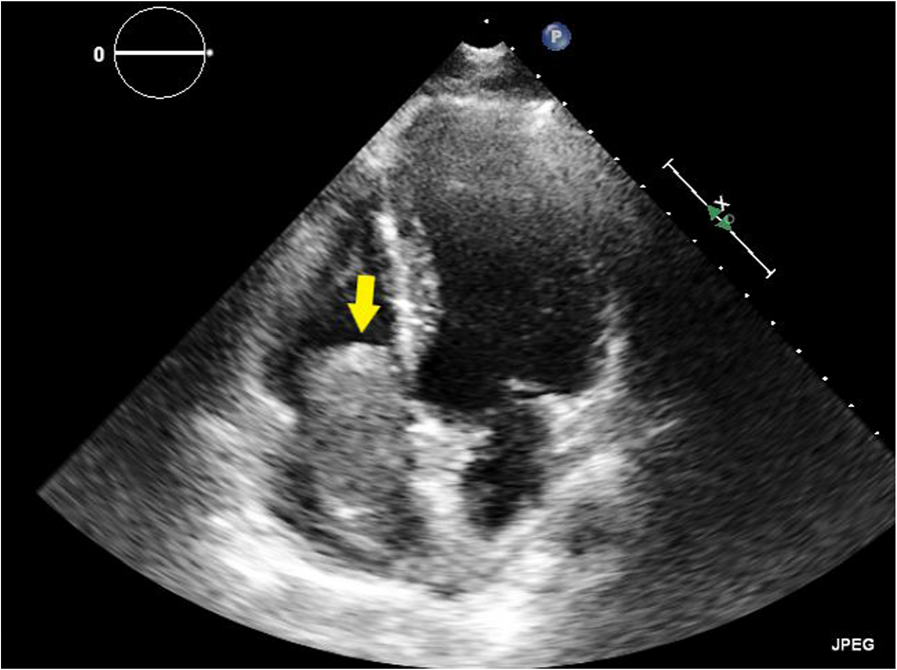
Transthoracic two-dimensional echocardiography, apical 4 chamber view showing a huge right atrial mass (about 8 × 7 cm), filling the whole cavity of the right atrium

**Fig. 3:**
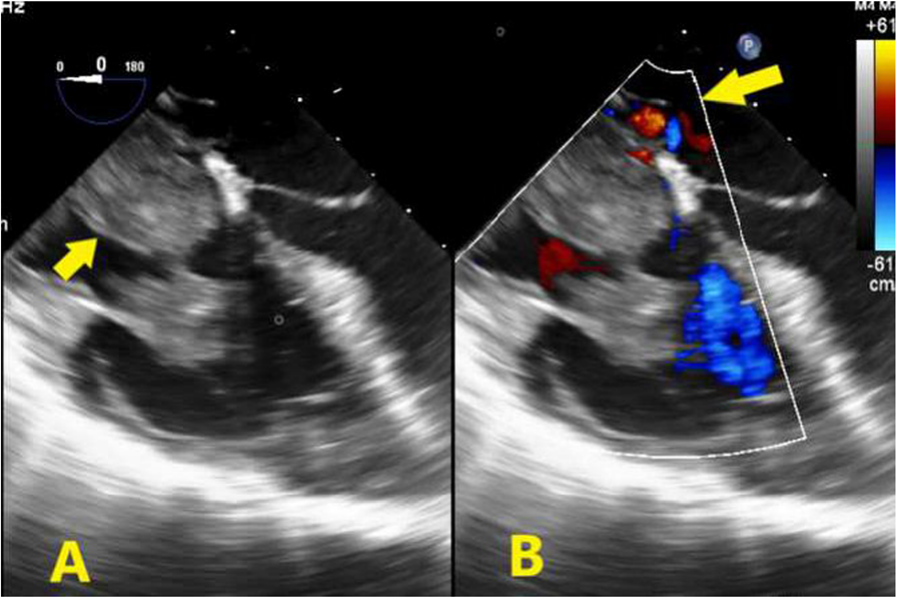
**a** Trans-esophageal two-dimensional echocardiography, mid-esophageal apical 4-chamber view showing a huge right atrial mass filling the whole cavity of the right atrium oscillating in and out of the right ventricle through the tricuspid valve. **b** Color Doppler showing patent foramen ovale

At the time of presentation, her blood pressure was 110/70 and her heart rate was 100/min. Her resting oxygen saturation was 88%. The rest of the physical examination was unremarkable apart from abdominal striae (steroids use). Her ECG showed normal sinus rhythm with no abnormalities (Fig. 1). Echocardiography showed a huge right atrial mass (about 8 × 7 cm), filling the whole cavity of the right atrium and oscillating in and out of the right ventricle through the tricuspid valve (Fig. 2, Additional file 1: Movie 1). The left ventricle appeared of normal dimensions and systolic function. The right ventricle appeared dilated with impaired systolic function. Laboratory findings showed marked thrombocytopenia (30.000) and severe renal impairment (creatinine 6.1 mg/dl and urea 176 mg/dl). Troponin was positive. Anti-thrombin III, lupus anticoagulant (LA), anti-cardiolipin (IgG, IgM), compliments 3 and 4, and proteins C and S were in the normal range. There was a hypoalbuminemia (albumin 1.9 mg/dl). Lower limb venous Duplex showed old canalized femoral deep venous thrombosis (DVT) with no evidence of acute DVT. Trans-esophageal echocardiography confirmed a huge right atrial mass that was extending from the inferior vena cava (IVC) and appeared to occupy the whole atrium. A small patent foramen ovale was also noticed in the inter-atrial septum (Fig. 3, Additional file 2: Movie 2). Differential diagnosis of the right atrial mass included thrombus (pulmonary emboli-in-transit), primary and metastatic cardiac tumors (e.g., right atrial myxoma), and vegetations on the tricuspid valve and intracardiac electrodes. The huge size, extension of the mass, and clinical context suggested that it was most probably a huge thrombus. The patient was admitted, IV heparin was initiated, and a heart team discussion was held regarding the best way of management. Urgent cardiac surgery to remove the right atrial mass was proposed as the best option for the patient taking into consideration its large size and risk of distant embolization. However, concerns were raised for the risk of peri-operative bleeding due to thrombocytopenia, the risk of renal failure, and the risk of recurring thrombosis post-operatively.

**Fig. 4:**
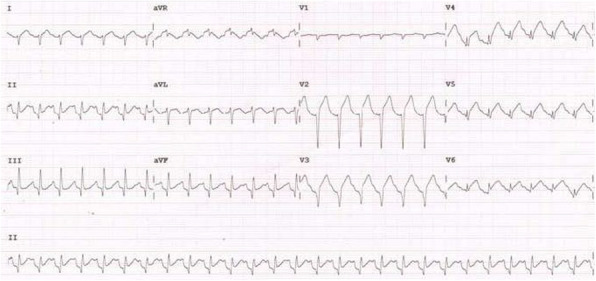
Twelve lead ECG showing ST segment elevation in anterior leads and sinus tachycardia

A few hours later, while the patient was in the intensive care unit (ICU), the patient arrested in asystole. Cardiopulmonary resuscitation (CPR) was immediately initiated, the patient was intubated, and she was resuscitated after two cycles of cardiopulmonary resuscitation. After the return of spontaneous circulation, she was vitally stable; the blood pressure was 140/90 and heart rate was 110/min. However, ECG showed ST-segment elevation in anterior chest leads (Fig. 4) and bedside echocardiography showed impaired left ventricle (EF of 40%) with akinetic mid and apical anterior septum and anterior wall, i.e., acute anterior STEMI, mostly due to paradoxical embolism. Recent updates were discussed, and a consensus was reached that surgery, despite the very high risk, will be the best management strategy.

**Fig. 5:**
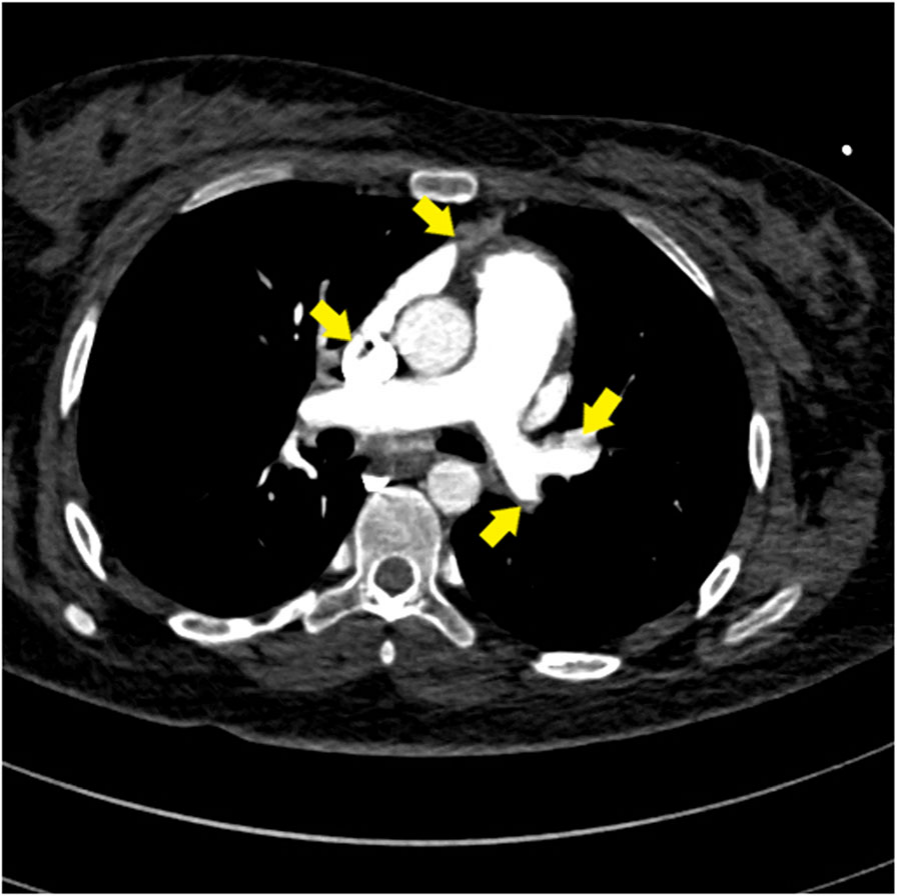
Multi-slice CT pulmonary angiography showing normal major pulmonary artery branches with occluded some peripheral pulmonary branches

As the patient was hemodynamically stable, we transferred her to the cath lab. Coronary angiography showed normal epicardial coronaries with mild haziness at mid segment of LAD artery with mild slow flow in the artery (canalized or dislodged embolus). The rest of the coronaries were normal (Additional file 3: Movie 3A, 3B). Multislice computed tomography (CT) pulmonary angiography was then performed (to exclude massive pulmonary embolism) and showed normal major pulmonary artery branches with occluded some peripheral pulmonary branches (Fig. 5). The inferior vena cava was clear of thrombi. No suspicious abdominal masses were visualized.

**Fig. 6:**
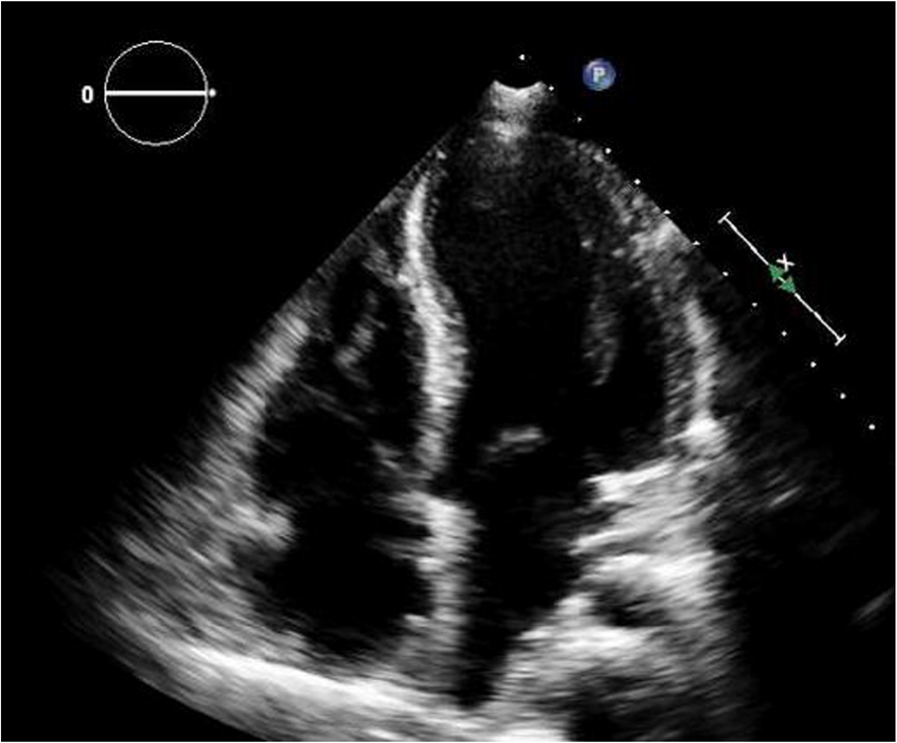
Post-operative transthoracic two-dimensional echocardiography, apical 4-chamber view showing no intra-cardiac masses

After a discussion with the family and explaining the high risk of mortality and morbidity, open-heart surgery was performed to excise the right atrial thrombus. Extra-corporeal membrane oxygenator (ECMO) was used as a support during surgery. At post-operative ICU, she was kept on IV steroids and had three sessions of hemodialysis. After 5 days, ECMO was disconnected and her vital signs stabilized. Two days later, she was extubated. A good urine output was maintained, the acid-base balance was normalized without the need for further dialysis, and creatinine and urea returned to the same levels as pre-operative. The platelets were stable at a level of 70,000. Oral anticoagulation using warfarin was initiated reaching an INR target of 2–3. Post-operative echocardiography showed borderline left ventricle (LV) contractility with regional wall motion abnormalities and mildly impaired right ventricle (RV) contractility with no evidence of mass residual (Fig. 6, Additional file 4: Movie 4). The patient was discharged on warfarin and oral steroids 3 weeks following surgery.

She returned to her daily activities with good functional capacity within 2 months after surgery. She was very happy and grateful to the whole team. She was referred to an immunologist and nephrologist to continue the treatment regimen of her underlying medical conditions.

## Discussion

Right heart thrombi (RHT) result either from migrating blood clots from deep venous thrombosis, or they may form in situ predominately due to atrial fibrillation.

While in-hospital mortality rates for patients presenting with acute pulmonary emboli reported to be about 2.5%, patients with RHT have an estimated mortality rate of about 28% and reaching to 80–100% in untreated cases [3]. As regards clinical presentation, patients with RHT were reported to have a rapid, risky, and even lethal outcome.

Trans-thoracic echocardiography (TTE) is the usual initial diagnostic tool for RHT. In 1989, the European Working Group on Echocardiography proposed a morphological classification and described three patterns of right heart thrombi [4, 5]. Type A thrombi, reported as the most common, are serpiginous worm-like in appearance, freely mobile within the heart chambers, and usually associated with deep vein thrombosis and pulmonary embolism. Type B thrombi are non-mobile, ovoid in shape, firmly attached to the chamber wall, and are believed to form in situ in association with underlying cardiac abnormalities. Finally, type C thrombi are rare, share a similar appearance to atrial myxoma, and are highly mobile. Trans-esophageal echocardiography (TEE) offers a better evaluation of the thrombus and should be considered when TTE is inconclusive; it can determine precisely its location especially in sites as the pulmonary artery or patent foramen ovale.

This lady was clearly diagnosed as having flaring of nephrotic syndrome manifested by hypoalbuminemia and prothrombotic tendency with the formation of right atrial thrombi. Nephrotic syndrome (NS) is defined by the presence of a nephrotic range of proteinuria, peripheral edema, hypoalbuminemia, hyperlipidemia, and increased risk of venous thromboembolic event (VTE) complications. Reduced serum albumin is an independent risk factor for thrombotic events in patients with NS. The increased propensity of thromboembolism in nephrotic patients is postulated to be a result of increased excretion of antithrombotic factors by the affected kidneys and increased production of pro-thrombotic factors by the liver. Most cases of VTE associated with NS reported in the literature have a preceding diagnosis of NS [6].

Immune thrombocytopenia (ITP) comprises a heterogeneous group of disorders characterized by autoimmune-mediated platelet destruction and impairment of platelet production [7]. Despite thrombocytopenia, previous studies suggested an increased risk of VTE in ITP as compared with the general population. Thromboembolic events have been reported in up to 8% of patients with ITP, suggesting that thromboembolism requires special precaution in this patient group. Thromboembolism can be caused by a disease (i.e., pro-thrombotic disease state) after the introduction of ITP therapies such as corticosteroids or can occur in association with other diseases [7]. On the other hand, venous thromboembolism (VTE) is an important and potentially life-threatening complication in focal segmental glomerulosclerosis (FSGS) [8]. The prevalence of venous thromboembolism is approximately 10% in FSGS patients with nephrotic syndrome. Hemoconcentration and relapse of the nephrotic syndrome were risk factors for the development of VTE in FSGS [8].

The impaired RV contractility in this case may be explained by previous repeated embolic showers to the pulmonary circulation and consequently elevated pulmonary artery systolic pressure. Moreover, the RV filling could be affected by the huge thrombus. Surgical embolectomy, mechanical unloading of the RV, and optimum post-operative anticoagulation contributed to the improvement of RV contractility postoperatively.

Treatment options for RHT include anticoagulation therapy, systemic thrombolysis, and surgical embolectomy. The optimal therapeutic approach is still a subject of debate. In the European Cooperative Study, the mortality rate was reported to be 60% for anticoagulated patients, 40% for those treated with thrombolytics, and 27% for those submitted to surgical procedures, which suggests the surgical approach to the most effective [5]. The largest meta-analysis to date was presented by Athappan et al. in 2015. It included 328 patients of whom 70 patients received anticoagulation, 122 patients received thrombolysis, and 120 patients had surgical embolectomy. The highest mortality rates (90.9%) were reported for patients who were left untreated. The mortality associated with anticoagulation alone was significantly higher than surgical embolectomy or thrombolysis (37.1% vs 18.3% vs 13.7%, respectively). In hemodynamically unstable patients, survival probability was higher in patients receiving thrombolysis (81.5%) than in patients treated with surgical embolectomy (70.45%), and both were far higher than anticoagulation alone (47.7%) [9]. Data on catheter-based interventions are few.

As regards surgical embolectomy, it has been specifically recommended in patients with right heart thrombi straddling the interatrial septum through a patent foramen ovale [10]. The limitations of this approach include the lack of availability of surgical expertise, an inherent delay while preparing for surgery, depressant effects of anesthetic drugs and cardioplegia, and the inability to remove co-existing peripheral pulmonary thrombi.

In the recent era, the widespread availability of multislice CT scanning facilitated rapid noninvasive detection of central pulmonary embolism amenable to embolectomy in many of patients [11], thus avoiding potential complications of conventional contrast pulmonary angiography. Rapid transport to the operating room is also a cornerstone of the management strategy. In critically ill patients with massive pulmonary emboli or floating right heart thrombi, a pulmonary embolism response team (PERT) approach [12], should always be considered where a multidisciplinary team involving a cardiologist, radiologist, cardio-thoracic surgeon, and radiologist shall determine the management strategy. In some instances, extracorporeal membrane oxygenation (ECMO) and/or surgical pulmonary embolectomy may be life-saving interventions.

On the other hand, thrombolytic therapy is a simple, rapid, readily available therapy, which can dissolve the thrombus at different locations (cardiac, pulmonary, and peripheral). Besides the risk of major bleeding, thrombolytic therapy may be associated with a postulated risk of clot fragmentation and migration, complete pulmonary embolization, or recurrent PE following the partial dissolution of the venous thrombus.

According to the European Society of Cardiology ESC 2019 guidelines of acute pulmonary embolism, our patient was considered to be at intermediate high risk (sPESI ≥ 1, plus impaired RV, and positive troponin). A higher risk was estimated due to the huge RA thrombus and the presence of PFO. Moreover, the patient sustained cardiac arrest and acute myocardial infarction due to paradoxical embolism. The arrest was initially suspected to be due to massive pulmonary embolism, but this was specifically excluded by multislice CT. So, the most probable explanation of the cardiac arrest is obstruction of the right ventricular inflow by the huge oscillating right atrial mass. Though our patient presented during the weekend, a time notorious for increased in-hospital mortality from many diseases including PE [13], and the availability of a well-trained multidisciplinary team was crucial for successful management. The care of hemodialysis and fluid balance was taken over by a nephrologist. Finally, the use of ECMO in this particular case was also a great aid in unloading both the stunned right and left ventricles in the early post-operative period.

## Conclusions

Right heart thrombi represent a quite challenging diagnosis that requires a precise determination of its source, morphology, anatomical extent, and clinical presentation. Nephrotic syndrome that presents with a very low serum albumin represents a prothrombotic condition with a high incidence of venous thromboembolism. Surgical embolectomy remains one of the golden modes of its management. A multidisciplinary team is the cornerstone for successful diagnosis, treatment, and follow-up of this risky clinical condition. Mechanical support of both ventricles may be needed in such critically ill patients and is highly deemed for speed recovery in the early post-operative period.

## Supplementary Information

**Additional file 1: Movie 1**: for Fig. 2. Transthoracic two-dimensional Echocardiography, apical 4 chamber view showing a huge right atrial mass (about 8x7 cm), filling the whole cavity of the right atrium.

**Additional file 2: Movie 2**: for Fig. 3. **(A)** Trans-oesphageal two-dimensional Echocardiography, mid-oesphageal apical 4 chamber view showing a huge right atrial mass filling the whole cavity of the right atrium oscillating in and out of the right ventricle through the tricuspid valve. **(B)** Color Doppler showing patent foramen ovale**. Movie 3:** showing haziness in proximal LAD with slow flow probably thrombus. **(A):** RAO Caudal view, **(B)** AP cranial

**Additional file 3: Movie 3:** showing haziness in proximal LAD with slow flow probably thrombus. **(A):** RAO Caudal view, **(B)** AP cranial

**Additional file 4: Movie 4**: for Fig. 6. Post-operative Transthoracic two-dimensional Echocardiography, apical 4 chamber view showing no intra-cardiac masses.

## Data Availability

All data generated or analyzed during this case are included in this published case (and its supplementary information files).

## Abbreviations

RHT: Right heart thrombi
APE: Acute pulmonary embolism
ECG: Electrocardiogram
ECMO: Extra-corporeal membrane oxygenator
PERT: Pulmonary embolism response team
PFO: Patent foramen ovale
ITP: Immune thrombocytopenic purpura
ICU: Intensive care unit
CPR: Cardiopulmonary resuscitation
TTE: Trans-thoracic echocardiography
TEE: Trans-esophageal echocardiography
LV: Left ventricle
RV: Right ventricle
ESC: European Society of Cardiology
